# The Complex Co-infections of Multiple Porcine Diarrhea Viruses in Local Area Based on the Luminex xTAG Multiplex Detection Method

**DOI:** 10.3389/fvets.2021.602866

**Published:** 2021-01-28

**Authors:** Ying Shi, Benqiang Li, Jie Tao, Jinghua Cheng, Huili Liu

**Affiliations:** ^1^Institute of Animal Husbandry and Veterinary Sciences, Shanghai Academy of Agricultural Sciences, Shanghai, China; ^2^Shanghai Key Laboratory of Agricultural Genetic Breeding, Shanghai Academy of Agricultural Sciences, Shanghai, China; ^3^Shanghai Engineering Research Center of Pig Breeding, Shanghai Academy of Agricultural Sciences, Shanghai, China

**Keywords:** porcine viral diarrhea, luminex xTAG multiplex detection method, epidemiology, co-infection, porcine epidemic diarrhea virus

## Abstract

The large-scale outbreaks of severe diarrhea caused by viruses have occurred in pigs since 2010, resulting in great damage to the pig industry. However, multiple infections have contributed to the outbreak of the disease and also resulted in great difficulties in diagnosis and control of the disease. Thus, a Luminex xTAG multiplex detection method, which was more sensitive and specific than general multiplex PCR method, was developed for the detection of 11 viral diarrhea pathogens, including PKoV, PAstV, PEDV, PSaV, PSV, PTV, PDCoV, TGEV, BVDV, PoRV, and PToV. To investigate the prevalence of diarrhea-associated viruses responsible for the outbreaks, a total of 753 porcine stool specimens collected from 9 pig farms in Shanghai during 2015–2018 were tested and the pathogen spectrums and co-infections were analyzed. As a result, PKoV, PAstV and PEDV were most commonly detected viruses in diarrheal pigs with the rate of 38.65% (291/753), 20.32% (153/753), and 15.54% (117/753), respectively. Furthermore, multiple infections were commonly seen, with positive rate of 28.42%. Infection pattern of the viral diarrhea pathogens in a specific farm was changing, and different farms had the various diarrhea infection patterns. A longitudinal investigation showed that PEDV was the key pathogen which was closely related to the death of diarrhea piglets. Other pathogens might play synergistic roles in the pathogenesis of diarrhea disease. Furthermore, the surveillance confirmed that variant enteropathogenic viruses were leading etiologic agents of porcine diarrhea, either mono-infection or co-infections of PKoV were common in pigs in Shanghai, but PEDV was still the key pathogen and multiple pathogens synergistically complicated the infection status, suggesting that controlling porcine diarrhea might be more complex than previously thought. The study provides a better understanding of diarrhea viruses in piglets, which will aid in better preventing and controlling epidemics of viral porcine diarrhea.

## Introduction

Viral diarrhea is a devastating disease in pigs in China and results in substantial economic losses to the pig industry worldwide (1). This disease increases the infection rate to piglets and mortality, which could reach up to 100% (2). Furthermore, multiplex infection and synergistic infection, which are commonly observed in clinic, pose a new challenge to disease diagnosis and control. The viruses that cause porcine diarrhea disease are diverse, including porcine epidemic diarrhea virus (PEDV) (3–5), porcine deltacorona virus (PDCoV) (6, 7), porcine transmissible gastroenteritis virus (TGEV) (8), porcine teschvirus (PTV) (9), bovine viral diarrhea virus (BVDV) (10), porcine rotavirus (PoRV) (11), porcine sapelovirus (PSV) (12), porcine kobuvirus (PKoV) (13–17), porcine astrovirus (PAstV) (11, 18–20), porcine torovirus (PToV) (21), and porcine sapovirus (PSaV) (22, 23), etc. An infection with any of these viruses can develop into similar clinical symptoms, including severe diarrhea and dehydration. However, it remains difficult to distinguish these pathogens in clinic.

Rapid turnaround time is important for diagnosis and infection control. Thus, the use of efficient methods for pathogen detection is necessary to ensure rapid turnaround time. Unlike the existing multiplex PCR methods that only focus on two or three targets and cannot be adapted to high-throughput testing. The recent Luminex xTAG assay has revolutionized the simultaneous detection and quantitation of multiple nucleic acids in a single reaction which are even available in small quantities (24). The tool had been gradually used in pathogen identification on account of its high specificity and sensitivity (25). Therefore, in this study, a multiple detection method for 11 diarrhea viruses based on Luminex technology was developed.

The precise data about the prevalence of multiple infections in porcine and wild boars have only been reported in a limited number of countries (26, 27). Moreover, the prevalence and multiplex infections of porcine diarrhea in Shanghai have not been studied thoroughly. Co-infection with various viruses makes preventing and curing diarrhea in pigs more complex. Although regular vaccine immunization has been strictly conducted, the high morbidity of diarrhea remains as a serious problem, which needs to be solved in time. Therefore, the epidemiology is needed to determine the prevalence in these circulating strains, in order to develop vaccination programs and establish a surveillance system. Thus, a surveillance of porcine viral diarrhea in Shanghai was conducted to better understand the frequency and evolution of these viruses in the field. Furthermore, the multiplex infection situation was analyzed, which will pave the way to improving the strategies in preventing and controlling virus infection in swine farms.

## Materials and Methods

### Specimens

A total of 753 porcine stool specimens were collected from 9 pig herds in Shanghai (Table 1), China from 2015 to 2018. Pigs of all ages were sampled and 1 to 3-week-old piglets were particularly collected. The intestinal samples used in this study were obtained from the dead piglets and the fecal samples were non-invasively collected immediately after excretion from diarrhea pigs and then submitted to our laboratory. Antibiotic treatment was invalid for all sampled pigs. Each sample was suspended in phosphate-buffered saline (PBS) containing 1,000 U/ml penicillin and 1,000 U/ml streptomycin and centrifuged at 12,000 r.p.m. for 10 min at 4°C. A portion of the suspension was used for RNA extraction, while the remaining supernatants were stored at −70°C.

**Table 1:** The details for porcine stool specimens.

**Year**	**District**	Farm serial number^*^	**Sample No**.	**Month**	**Status**
2015	Chongming	1	48	11	Diarrhea
	Pudong	2	15	12	Death
2016	Jinshan	3	41	3	Diarrhea
		3	12	12	Diarrhea
		4	20	12	Diarrhea
		5	30	12	Diarrhea
		6	23	12	Diarrhea
		7	25	12	Diarrhea
	Chongming	1	101	12	Diarrhea
		1	49	1	Diarrhea
	Jiading	8	30	12	Diarrhea
		9	25	12	Diarrhea
2017	Pudong	2	59	1	Diarrhea
	Chongming	1	40	2	Diarrhea
2018	Chongming	1	235	3	Diarrhea

A total of 753 porcine stool specimens were collected from 9 pig herds in Shanghai, China from 2015 to 2018. Pigs of all ages were sampled and 1 to 3-week-old piglets were particularly collected. The intestinal samples used in this study were obtained from the dead piglets and the fecal samples were non-invasively collected immediately after excretion from diarrhea pigs and then submitted to our laboratory.

* *The same serial number represents the specimens were collected from the same farm*.

### Establishment of Luminex xTAG Multiplex Detection Method

Systems using xTAG Technology perform discrete assays on the surface of color coded beads known as microspheres, which are then read in a compact analyzer. Using multiple lasers and high-speed digital-signal processors, the analyzer reads multiplex assay results by reporting the reactions occurring on each individual microsphere. The magnetic microspheres pre-coupled with anti-TAG oligo-nucleotides capture the matching oligo-nucleotide presented on the 5′ end of PCR amplicons. A Luminex fluorescence reader is then used for analytical measurements of bead types. According to the conserved sequences in GenBank, DNAStar and Oligo7 software were used to design the PCR primer pairs of 11 pathogens (Table 2). The M gene of PToV, M gene of PDCoV, RDRP gene of PAstV, 3D gene of PKoV, RDRP gene of PSV, 3D gene of PTV, RDRP gene of PsaV, 5′ URT gene of BVDV, M gene of PEDV, N gene of TGEV, and VP6 gene of PoRV were as target genes. Spacer C12 was added between the 5′ end of all upstream primers and the 3′ end of the anti-TAG sequence, and the 5′ end of all downstream primers was modified with biotinylated (biotin-) tags. The biotin-tagged amplifications and TAG microspheres were hybridized after selecting TAG-microspheres complementary to anti-TAG sequences. The hybridization product for liquid-phase chip detection was obtained to detect the pathogen, and the quantitation of different pathogen was measured according to the mean fluorescence intensity (MFI) value. The specificity and sensitivity of Luminex xTAG assay method were analyzed, and the optimal conditions of hybridization system and reaction were also optimized.

**Table 2:** The PCR primer pairs of 11 pathogens.

**Virus**	**Gene**	**Sequence (5^**′**^-3^**′**^)**
PToV	M	AACTTTCTCTCTCTATTCTTATTT/iSpC12/AATTGCTTATTGGTGGCTTC
		Biotin-AGCDATTTGRGCDGCATTC
PDCoV	M	CTATCATTTATCTCTTTCTCAATT/iSpC12/CGCAGTTTTCATTGTGTCCA
		Biotin-CCTGTGGCGGATTTCTAACT
PAstV	RDRP	CAATTTACATTTCACTTTCTTATC/iSpC12/CCTTWCCCCACTGATGAAGA
		Biotin-CCTGTCCATCTGCCTTTCTGT
PKoV	3D	TCATCACTTTCTTTACTTTACATT/iSpC12/CGCCGTTCACTCTTTGTCC
		Biotin-ACCAAGCAGCATCCTACCAG
PSV	RDRP	AATTTCTTCTCTTTCTTTCACAAT/iSpC12/CTGGACTGGGCCTATACT
		Biotin-TYAGGTACACACGGGCTC
PTV	3D	ACACTCATTTAACACTATTTCATT/iSpC12/TGCAGCTCTTTCACATTTRGA
		Biotin-ACTTGTATGAGGCCCATCG
PSaV	RDRP	ACAAATATCTAACTACTATCACAA/iSpC12/GTGGCAACGTACAAYGCRTGG
		Biotin-GCCTCCATCACGAACACT
BVDV	5′URT	ATACTTTACAAACAAATAACACAC/iSpC12/AGCCATGCCCHTAGTAGGAC
		Biotin-CCTCTGCWRCACCCTATC
PEDV	M	CTTTCTCATACTTTCAACTAATTT/iSpC12/GTCAAGATGGCTATTCTATGG
		Biotin-ACCAAGAATGTGTCCTGCG
TGEV	N	TTAACAACTTATACAAACACAAAC/iSpC12/CAAAGGAATAGGTAACAGGG
		Biotin-ATCTGCATGAGGTCCAGT
PoRV	VP6	CATAATCAATTTCAACTTTCTACT/iSpC12/GGATTACTTGGYACTACTTT
		Biotin-AATGATGCTGAYTATGGAAGT

*The M gene of PToV, M gene of PDCoV, RDRP gene of PAstV, 3D gene of PKoV, RDRP gene of PSV, 3D gene of PTV, RDRP gene of PsaV, 5' URT gene of BVDV, M gene of PEDV, N gene of TGEV and VP6 gene of PoRV were as target genes. Spacer C12 was added between the 5' end of all upstream primers and the 3' end of the anti-TAG sequence, and the 5' end of all downstream primers was biotinylated (Biotin-) tag; selecting TAG microspheres complementary to anti-TAG sequence*.

### Multiplex PCR

According to manufacturer's instructions, total RNA was extracted from the fecal supernatant using TRIzol reagent (Life Technologies, Gaithersburg, MD, USA). Viral RNA was converted to cDNA using reverse transcriptase M-MLV (TaKaRa, Dalian, China) in a final volume of 20 μL containing 5 μL RNA, 4 μL 5 × RT buffer, 2 μL dNTPs (10 mM), 2 μL 10 μM random primer, 0.5 μL M-MLV reverse transcriptase, 0.5 μL RNase inhibitor (TaKaRa, Dalian, China), and 6 μL diethylpyrocarbonate (DEPC)-treated water. The reaction was incubated at 42°C for 1 h, followed by incubation at 70°C for 15 min. Afterwards, the cDNA was screened using the method we had established. The amplification steps were: 95°C for 4 min and 30 cycles of 95°C for 30 s, 55°C for 30 s, and 72°C for 30 s and a final step of 10 min at 72°C. All samples were tested in triplicate and all assays were run with positive and negative controls.

### Hybridization and Luminex Analysis

xTAG microspheres were purchased from Luminex (Austin, TX, United States) and suspended in tetramethylammonium chloride as outlined in the manufacturer's protocol. Nucleic acid hybridization was carried out in a 100 μL volume including 5 μL of amplified product, 2 0μL of the working microsphere mixture that contained 2,500 beads of each target-specific microsphere and 75 μL Streptavidin-phycoerythrin (SAPE). Hybridization was carried out at 37–45°C min for 25–45 min to explore the optimal hybridization reaction conditions. The products were analyzed using the Luminex 200 reader immediately after hybridization and the results were expressed as MFI.

### Longitudinal Investigation of Enteric Pathogens

A longitudinal follow-up study was setup between February and April in 2017. To warrant the health status of the pig stock, entrance to the farm was strictly regulated, and sampling was performed by the farmer. Detailed instructions and sampling materials were provided to the farmer. A dry cotton rectal swab was collected from each individual pig with diarrhea and was placed immediately in 2 ml of viral transport medium (phosphate buffered saline containing 1,000 U/ml penicillin and 1,000 U/ml streptomycin) and stored at −20°C. The farmer was asked to mark the tube of each sample for diarrheic signs. Every week, samples were collected from the farm and transported to the laboratory for pathogen detection.

## Results

### Establishment of Luminex xTAG Multiplex Detection Method

**Figure 1:**
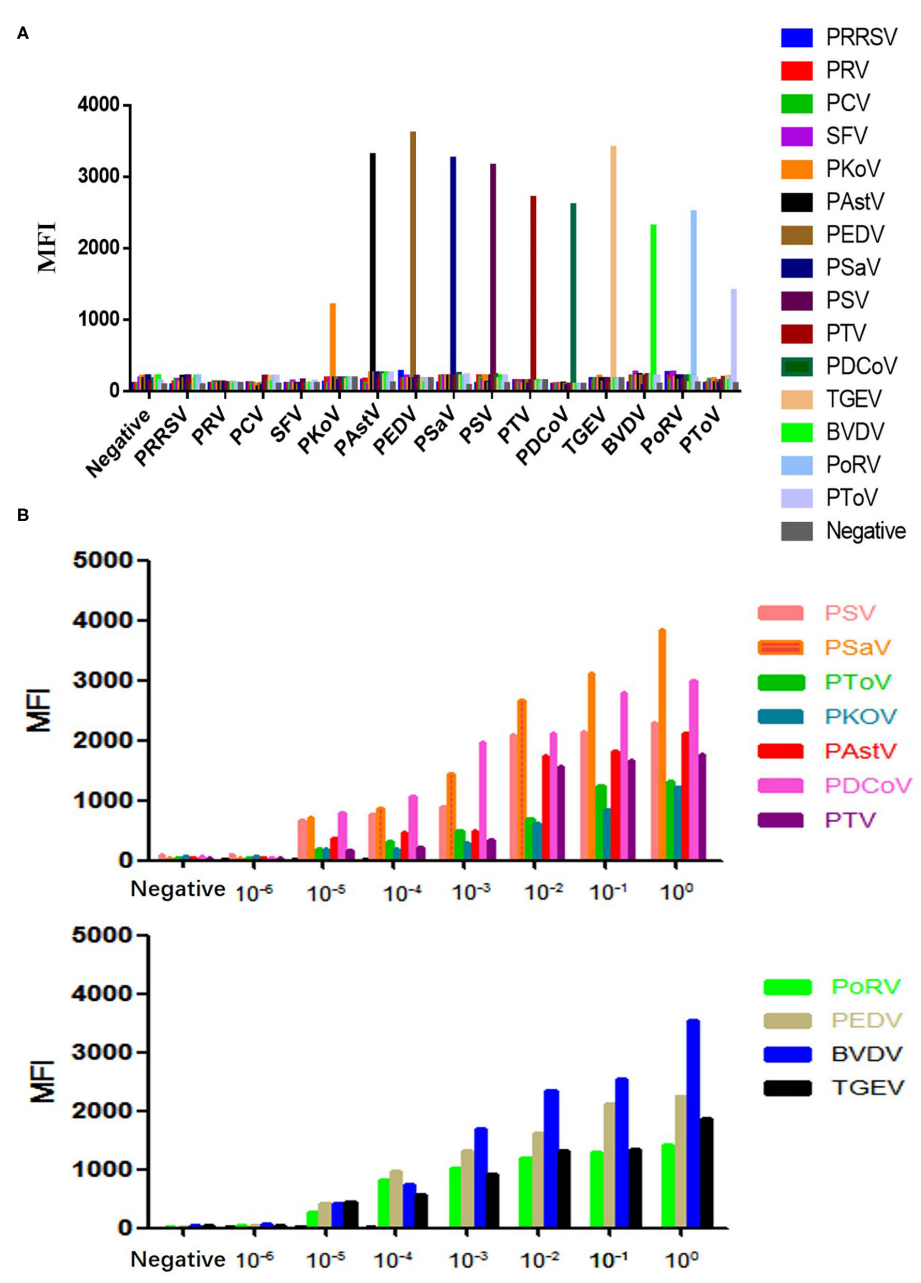
The specificity and sensitivity detection of the Luminex test. **(A)** The specificity detection. **(B)** The sensitivity detection.

Based on a singular detection system, a Luminex xTAG multiplex detection method for the simultaneous detection of 11 diarrheal viruses was established. The optimal hybridization system and reaction condition were as follows: 20 μL microsphere working solution, 5 μL PCR amplification product, and 75 μL SAPE report buffer; the results of optimal hybridization temperature was 42°C, and the best hybridization time was 30 min. The specificity of Luminex xTAG assay method was tested, which showed that each primer pair had good specificity and there was no cross-reaction between the primer pairs (Figure 1A). The sensitivity test of the Luminex xTAG detection method (Figure 1B) showed that the minimum detection limit was PTV in 3.12 × 10^3^ copies/μL, PKoV in 2.92 × 10^3^ copies/μL, PDCoV in 2.79 × 10^3^ copies/μL, PSV in 3.37 × 10^3^ copies/ μL, PSaV in 2.7 × 10^3^ copies/μL, PAstV in 3.02 × 10^3^ copies/μL, PToV in 2.65 × 10^3^ copies/μL, PoRV in 2.57 × 10^3^ copies/μL, PEDV in 1.74 × 10^3^ copies/μL, BVDV in 2.41 × 10^3^ copies/μL, and TGEV in 2.75 × 10^3^ copies/μL, respectively. The sensitivity of the established method with the traditional multiple RT-PCR method were also compared (Supplementary Figure 2). The minimum detection rate was at least 10 times higher than the traditional multiplex PCR method.

**Figure 2:**
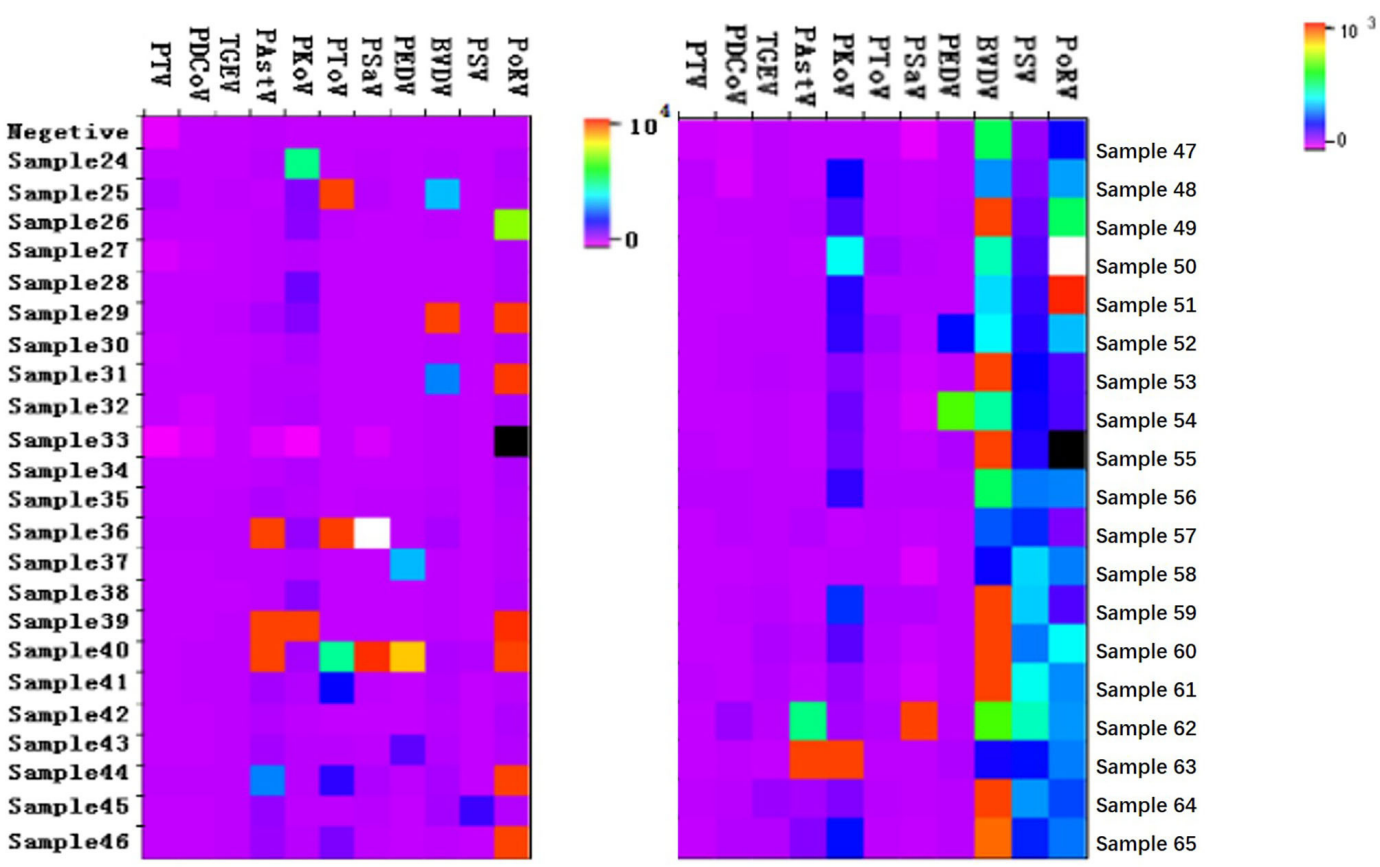
The Heatmap for quantitative results of some samples. The porcine stool specimens were tested by the Luminex xTAG multiplex detection method. Only part data were present in the figure.

The pathogen composition in each sample could be estimated intuitively. The concentration of different pathogens in each sample was presented as different colors in a heat-map (Figure 2). Therefore, different colors represent different concentrations of pathogens. The closer the color was to black, the lower concentration was present, and the closer the color was to red, the higher concentration was present in the sample. In addition, the predominant viruses in the multiplex infection samples could be speculated according to quantitative analysis. Therefore, in virtue of the quantitative analysis, the detection results will be more clearly visible and targeted prevention or therapy may be carried out in the pigsty, paving the way to instructing the clinical production.

### Viral Pathogens Infected in Diarrhea Stools Were Diversified

**Figure 3:**
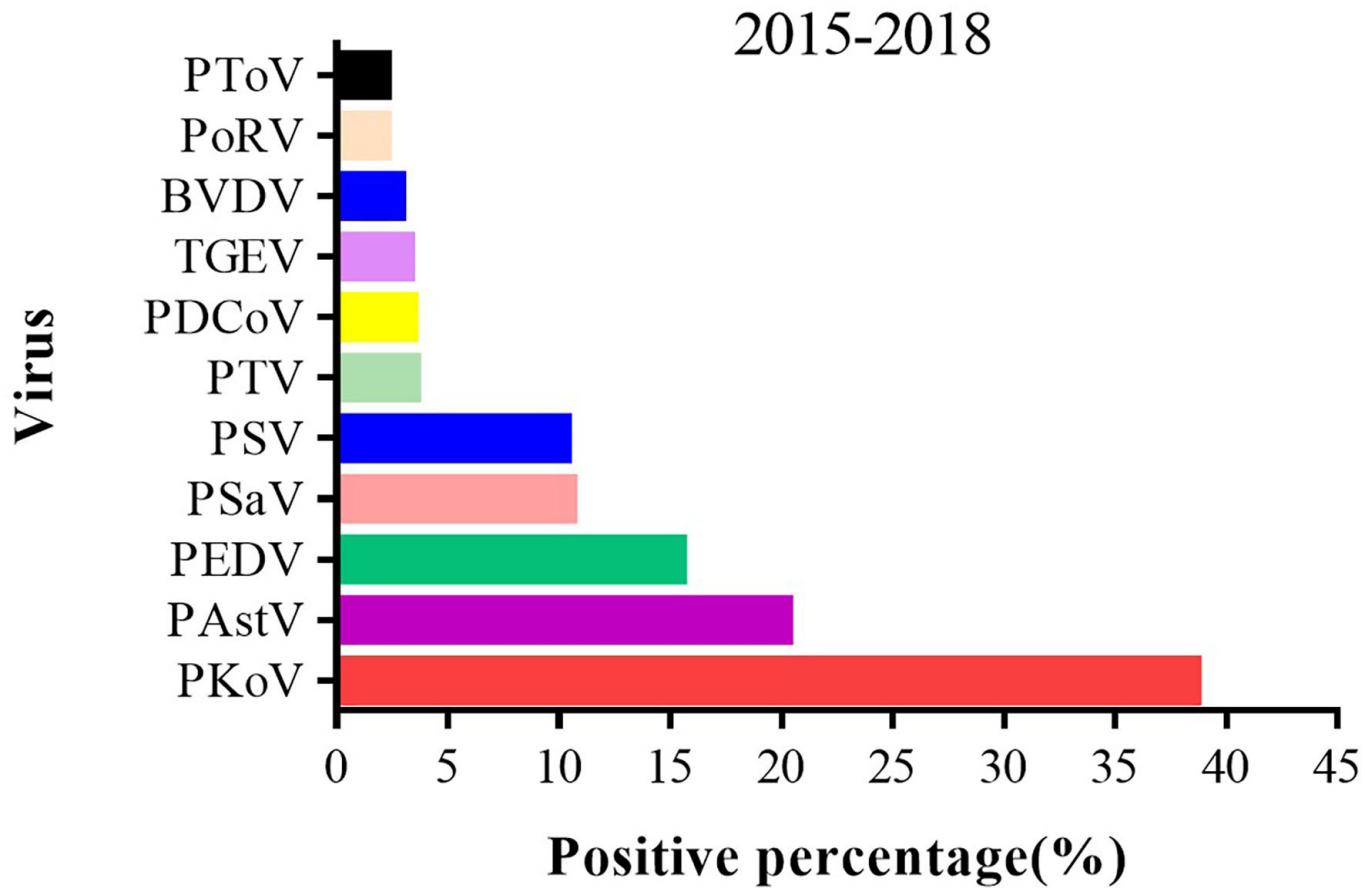
The positive percent of different enteric viruses from 2015 to 2018 in Shanghai. A total of 753 porcine stool specimens from 5 districts in Shanghai were detected by multiplex RT-PCR method.

A total of 753 porcine stool specimens from five districts in Shanghai were detected using the established Luminex xTAG multiplex detection method. Of the 753 diarrhea samples, PEDV was found in 15.54% (117/753), PKoV in 38.65% (291/753), PAstV in 20.32% (153/753), PSaV in 10.62% (80/753), PSV in 10.36% (78/753), PTV in 3.59% (27/753), PDCoV in 3.45% (26/753), TGEV in 3.32% (25/753), BVDV in 2.92% (22/753), PoRV in 2.26% (17/753), PToV in 2.26% (17/753), respectively (Figure 3). Therefore, complicated pathogen composition existed in clinic, which emphasized the importance of monitoring the fluctuant infection spectrum to guide the clinical production.

### Multiple Infections Were Commonly Seen in Clinic

**Figure 4:**
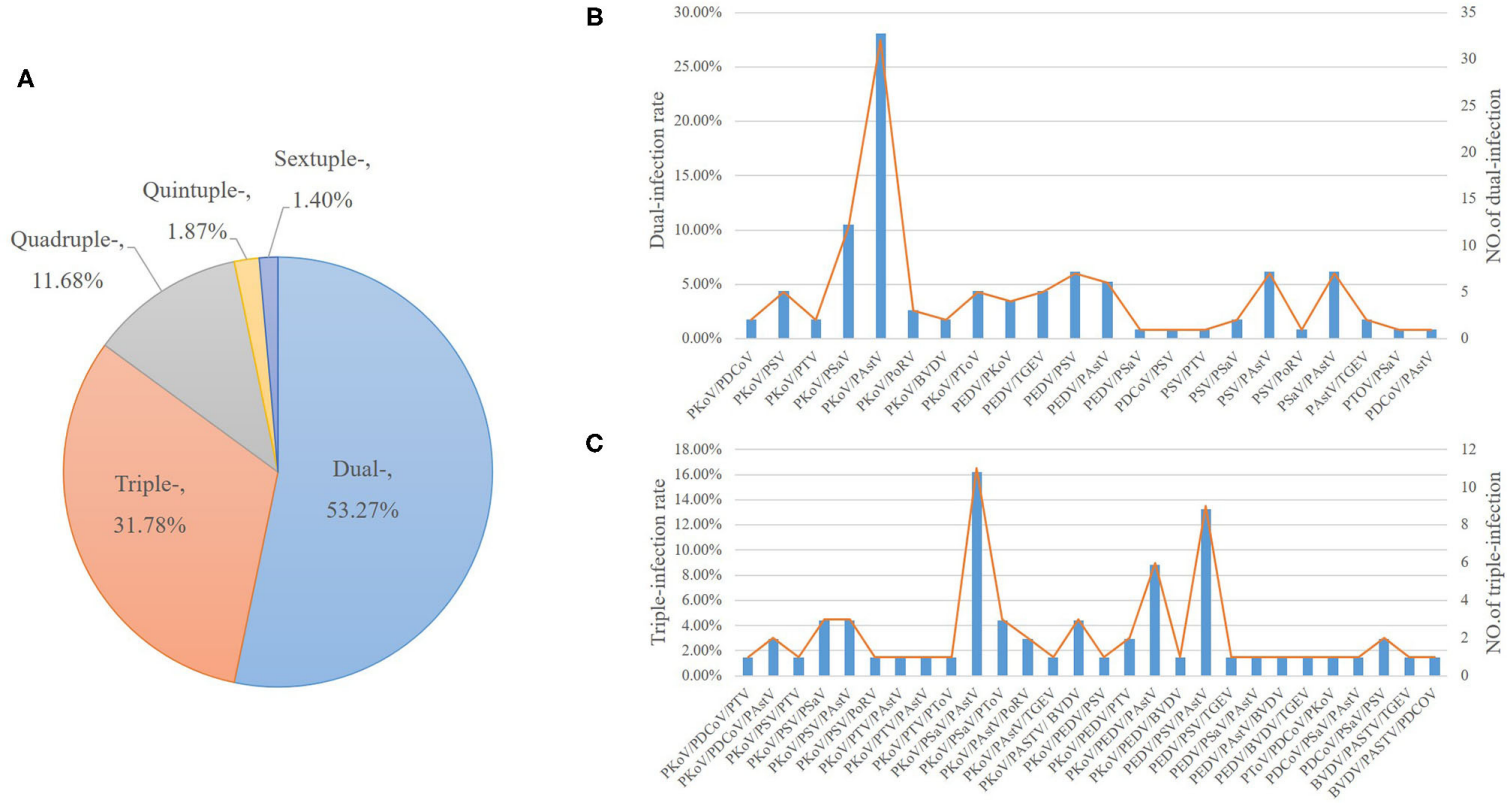
The multiple infections of different enteric viruses. Various combinations of co-infections were identified. **(A)** The percent of multiple infections of different enteric viruses. **(B)** The positive percent (left Y axis) and positive numbers of dual-infections (right Y axis). **(C)** The positive percent (left Y axis) and positive numbers of triple-infections.

A total of 214 co-infections were identified, with positive rate of 28.42%. Among the samples positive for the 11 enteric viruses, 53.27% (114/214) of samples had at least two pathogens, and 31.78% (68/214) of samples had three different pathogens, while the positive rate of quadruple- and quintuple-infection was 11.68% (25/214) and 1.87% (4/214), respectively. Most surprisingly, six viral diarrhea pathogens (1.40%, 3/214) were simultaneously detected from one diarrhea stool, indicating a complex diarrhea pathogen infection pattern and pathogenesis in clinic (Figure 4A).

Among the samples positive for the 11 enteric viruses, PKoV exhibited high co-infection rates, ranging from 1.75 to 28.07% (Figure 4B). Of the 114 dual-infections, 67 samples were PKoV-positive (58.77%) and 23 samples were PEDV-positive (20.18%). The co-infection of PKoV-positive samples with PAstV, PSaV, and PEDV reached 28.07, 10.53, and 3.51%, respectively (Figure 4B). Of the 117 PEDV-positive samples, dual-infection with PKoV, PSV and PAstV reached 3.51, 6.14, and 5.26%, respectively (Figure 4B). Of the 68 triple-infections, 44 samples were PKoV-positive (64.71%) and 23 samples were PEDV-positive (33.82%), while 10 (14.71%) samples were co-infected with PKOV, PEDV and other diarrhea viruses (Figure 4C). Among the 25 quadruple-infections, 12 samples were PKoV-positive (48%) and 6 samples were PEDV-positive (24%). All the quintuple and sextuple-infections were positive with PKoV and PEDV. There were no cases of co-infection with any combination of PEDV, TGEV, and PRoV. Interestingly, co-infections with PEDV and PKoV accounted for 24.79% (29/117) of the PEDV positive samples. PKoV was always one of the co-infecting viruses, which indicated that PKoV might play synergistic roles in the pathogenesis of diarrhea disease.

### Different Farms Showed Variable Infection Spectrum

**Figure 5:**
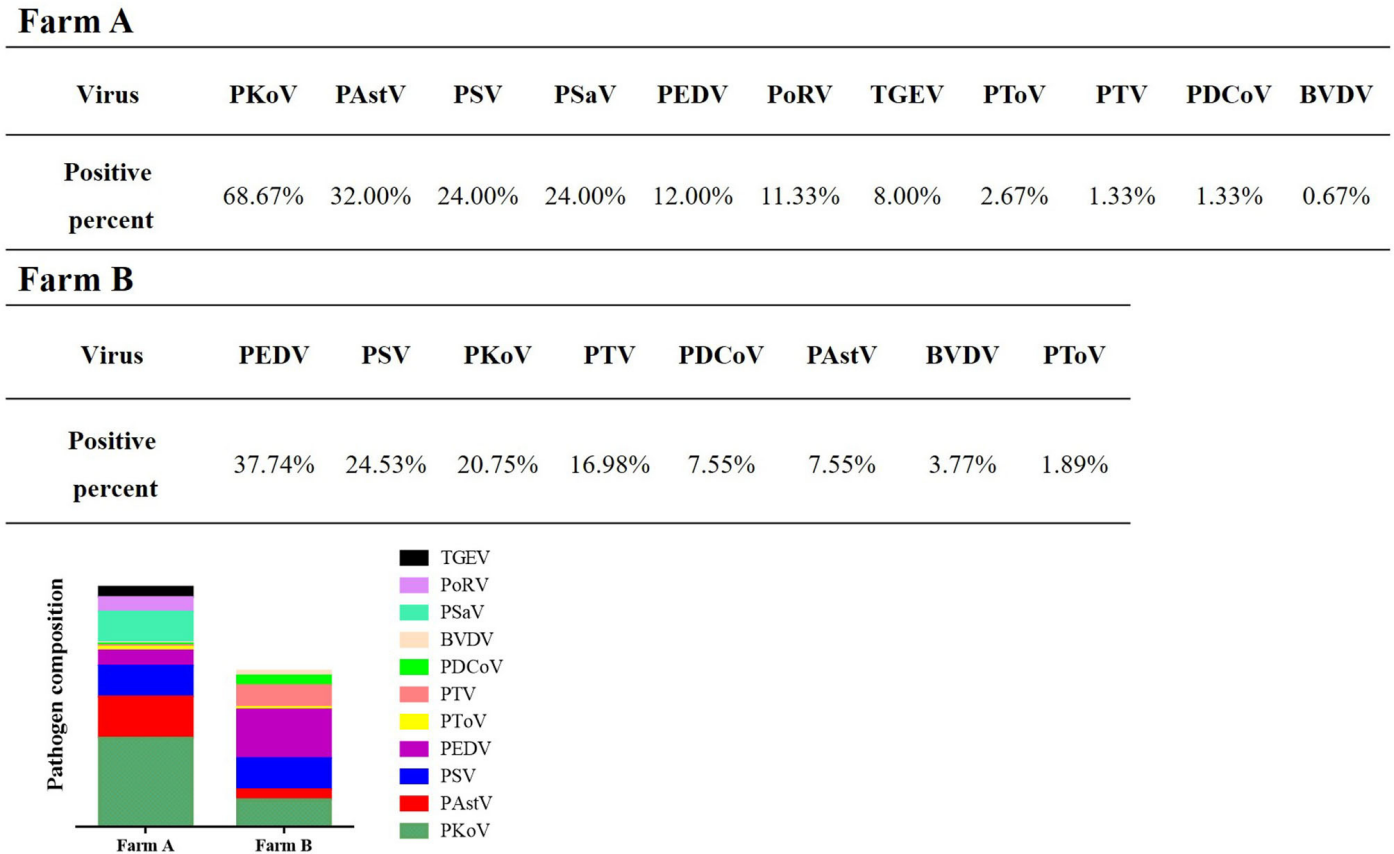
Detection of enteric viruses in different farms. The composition of enteric pathogens among different farms was analyzed.

**Figure 6:**
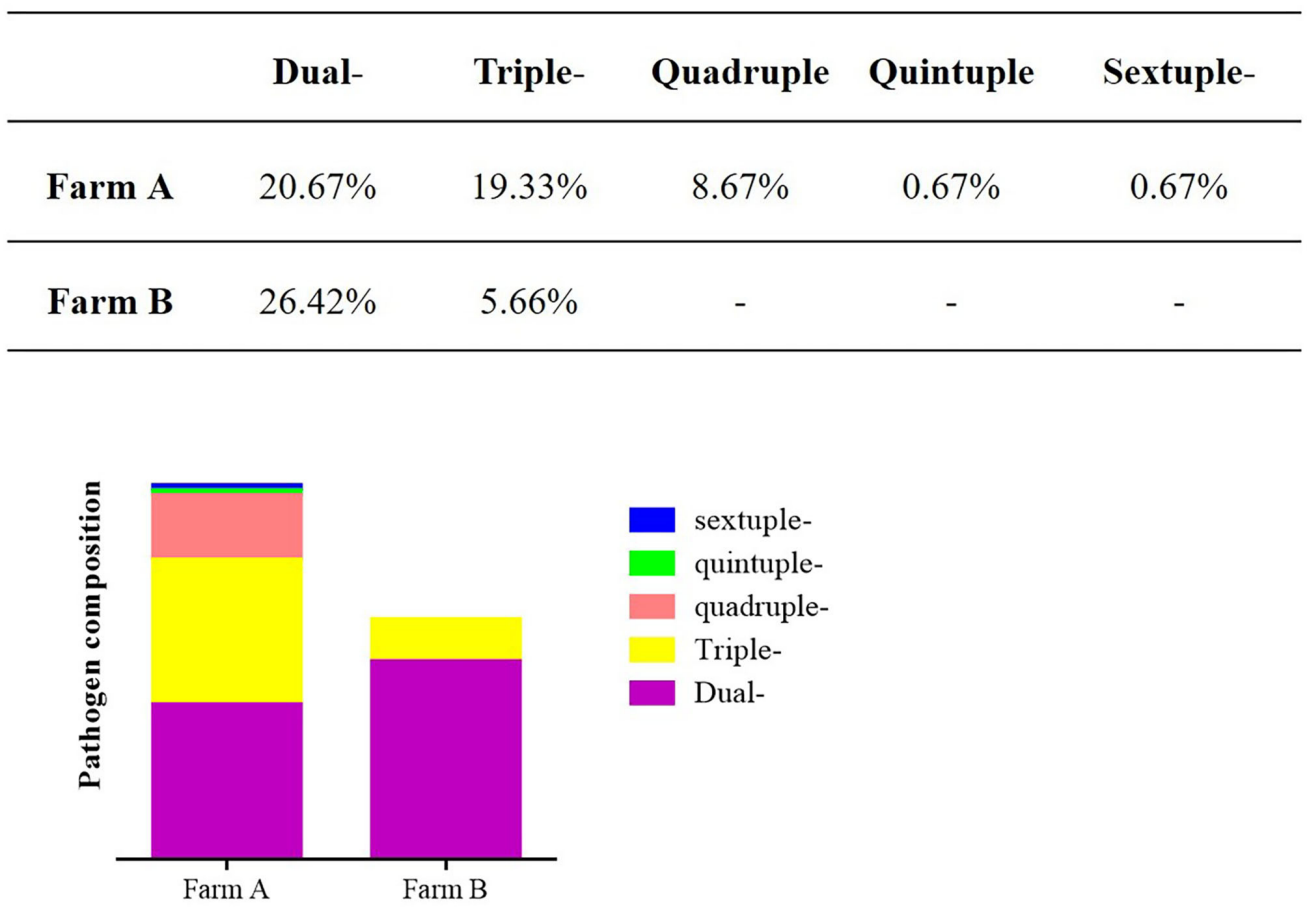
The co-infections of different farms. Different co-infections were analyzed in different farms.

The composition of enteric pathogens among different farms was analyzed (Figures 5, 6). In farm A, eleven kinds of pathogens were detected including multiple co-infections (dual-, triple-, quadruple- quintuple-, and sextuple-infection). In farm B, only eight kinds of pathogens were identified including dual- and triple-infection. It was concluded that the pathogen composition of a specific farm was unique. Therefore, different prevention and control measures should be carried out based on the monitored pathogens.

### Viral Infection Spectrum in a Specific Farm

**Figure 7:**
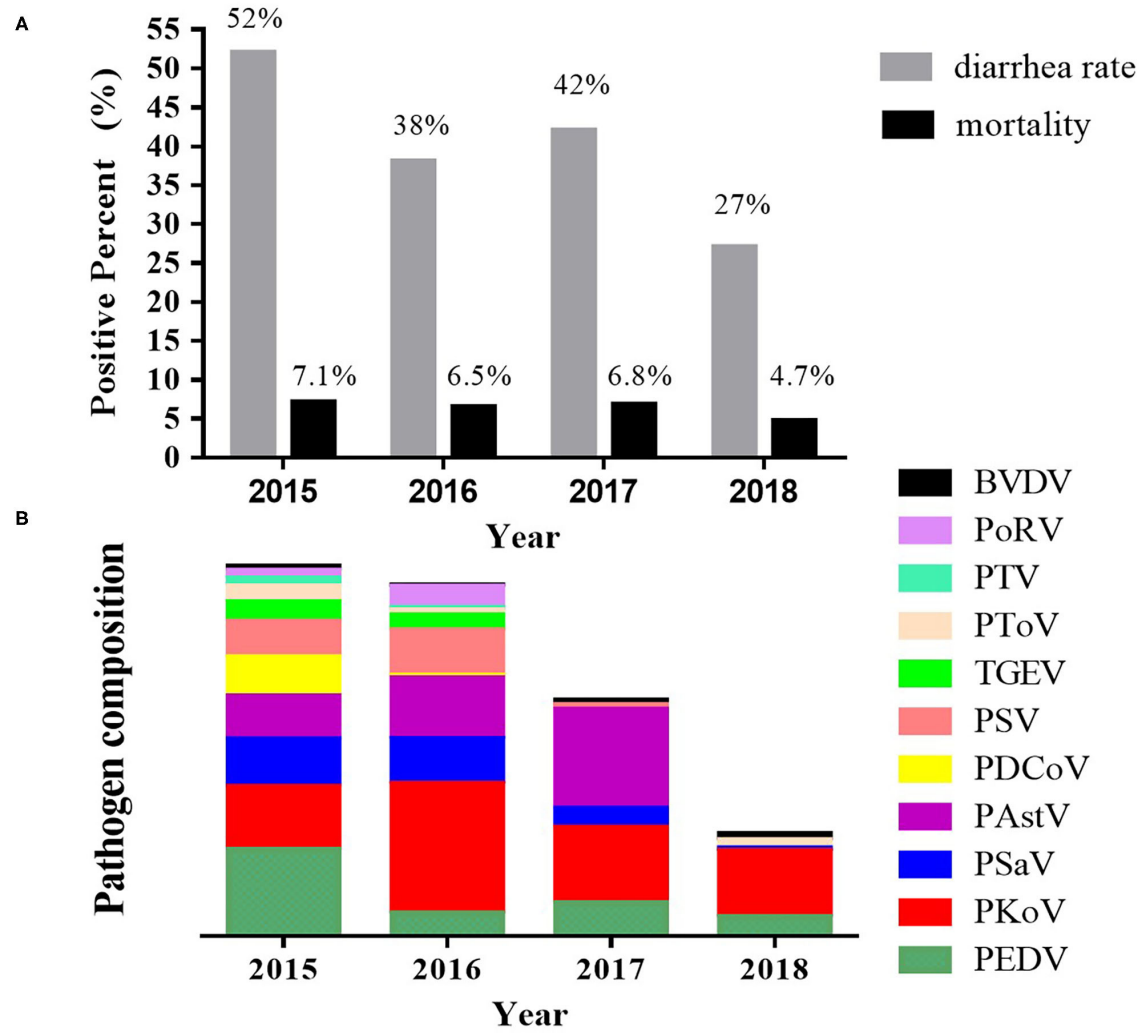
Pathogen spectrum of a specific farm from 2015 to 2018. The porcine stool specimens were detected every year from 2015 to 2018. It was observed that the prevalence of the viral diarrhea pathogens also changed over time. **(A)** The diarrhea rate and mortality. **(B)** The pathogen composition in different years.

In tracking the annual viral diarrhea tests in one farm from 2015 to 2018, it was observed that the prevalence of the viral diarrhea pathogens also changed over time (Figure 7). In 2015, PEDV had the highest positive rate of 45.83%, while the second highest was PKoV (33.33%). Accordingly, high diarrhea rate (52%) and mortality (7.1%) were observed. In 2016, PKoV became the most popular pathogen with a particularly high positive rate of 68.67%, and this was closely followed by PAstV (32%) and PSV (24%). The PEDV positive rate was merely 12%, accordingly, diarrhea rate decreased to 38% and mortality to 5.5%. In 2017, PAstV had the highest detection rate of pathogens (52.50%), followed by PKoV (40%) and PEDV (17.5%), while the diarrhea rate and mortality became 42 and 6.8%, respectively. In 2018, PKoV had the highest detection rate of pathogens (35.03%), followed by PEDV (10.19%), while the diarrhea rate and mortality became 27 and 4.7%, respectively. Taken together, these results do indicate that the fluctuations of diarrhea rate and mortality were accordance with the tendency of PEDV positive rate, while high rate of PKoV or PAstV did not necessarily correspond to high diarrhea rate and mortality.

### Longitudinal Investigation Showed Dominant Infection Agents

A longitudinal follow-up study was setup between February and April in 2017 to identify the dominant pathogen. A total of 173 diarrhea stools were collected and detected. As a result, 11 viral diarrhea pathogens were detected, and the positive detection rate of PEDV, PKoV, PoRV, PSV, BVDV, PSaV, PToV, PAstV, TGEV, PDCoV, and PTV was 32.37, 26.01, 14.45, 6.94, 6.36, 11.56, 2.89, 26.01, 3.47, 0.58, and 1.73%, respectively (Table 3). A total of 60 co-infections were identified, with positive rate of 34.68% (60/173) (Table 4). Among the 11 enteric viruses, 48.33% (29/60) of samples had at least two pathogens, and 31.67% (19/60) of samples had three different pathogens, while the positive rate of quadruple- and quintuple-infection was 10.00% (6/60) and 5.00% (3/60), respectively. Six viral diarrhea pathogens were simultaneously detected from one diarrhea stool (5.00%, 3/60). Of the 56 PEDV-positive samples, co-infection rate with PKoV and PoRV reached 16.07% (9/56) and 5.36% (3/56), respectively. PKoV was always one of the co-infecting viruses, however, the viral load was not extremely high according to the quantitative analysis.

**Table 3:** The detection of various pathogens from a longitudinal follow-up study in 2017.

**Virus**	**PEDV**	**PKoV**	**PAstV**	**PoRV**	**PSaV**	**PSV**	**BVDV**	**TGEV**	**PToV**	**PTV**	**PDCoV**
Positive numbers	56	45	45	25	20	12	11	6	5	3	1
Positive percent	32.37%	26.01%	26.01%	14.45%	11.56%	6.94%	6.36%	3.47%	2.89%	1.73%	0.58%

*One hundred seventy three diarrhea samples collected in 2017 were further quantitatively detected using the Luminex xTAG multiplex detection method*.

**Table 4:** The co-infections of clinical samples from a longitudinal follow-up study in 2017.

**Complex infection**	**Positive No**.	**Positive percent %**
Dual-	29	16.76
Triple-	19	10.98
Quadruple-	6	3.47
Quintuple-	3	1.73
Sextuple-	3	1.73

*A total of 173 samples were detected. Different multiplex infections had been shown in the table*.

The morbidity and mortality rate of the farm during sampling was 57 and 5.23%; even vaccinated suckling piglets were not spared. The infected piglets predominantly showed yellow watery stools, weight loss, and death from dehydration. Interestingly, single PEDV infection or co-infection of PEDV with other diarrhea viruses was identified in all the dead piglets. One piglet infected with 6 kinds of diarrhea viruses including PEDV, finally died. While another piglet infected with six other viruses was still alive with great weight loss. All in all, PEDV is still the key pathogen which was closely related to the death of diarrhea piglets. Other pathogens might play synergistic roles in the pathogenesis of diarrhea disease.

According to the present surveillance results, relative proposals and solutions were put forward and carried out to control the emerging diarrhea and to prevent later potential occurrence.

## Discussion

Porcine viral diarrhea disease seriously endangers the development of the pig industry, and leads to significant economic losses for pig farmers worldwide (28). Clinically, the complexity of the disease has increased. In some cases, multiplex infections with two or more viruses are common, which seriously interfere with the clinical diagnosis (6, 7, 26, 27, 29). It has been speculated that the incidence of diarrhea would decline due to the vaccine prevention of PEDV, TGEV, and PoRV triplets. However, diarrhea continued to threaten pig farms. Beside these three traditional porcine viral diarrhea pathogens (PEDV, TGEV, and PoRV), other viral diarrhea pathogens have also been reported in recent years (9–12, 17, 21, 23, 30). In particular, the situation of multiplex infections has become more serious, resulting in increased pressure in the prevention and control of porcine diarrhea. Although the correlation between emerging viruses and diarrhea has not been clearly discussed, these co-infections have indeed enhanced the severity of diarrhea in the present study. Therefore, in order to accurately differentiate the infections in clinical specimens and prevent the transboundary spread of porcine viral diarrhea disease, it is necessary to conduct pathogen monitoring in clinical production.

Currently, PCR-based methods have been proven to be convenient and highly sensitive for detecting porcine diarrhea-associated viruses (2, 31). The multiple PCR method for testing 4 or 7 kinds of diarrhea pathogens has been established in the laboratory of the investigators, and was applied for clinical detection (31, 32). However, complicated multiplex infections require a more accurate detection method. Therefore, we further developed a Luminex xTAG high-throughput detection method for viral diarrhea pathogens in pigs, which has the advantages of high flux, wide range of detection, and small sample quantities. Furthermore, this is suitable for the large-scale screening of clinical samples, and is especially suitable for the multiplex infection detection of samples. Using Luminex xTAG technology, the standard curves for the above 11 diarrheal pathogens were established, and the content of each pathogen was calculated by measuring the MFI value of each sample. By analogy, the viral load can be determined for each sample, and finally, the pathogen with the highest risk of infection in each pig farm was analyzed, which would be helpful to guide the formulation of immunity and control measures in pig farms. In addition, this can also intuitively identify the maximum level of pathogens in each sample based on the cluster analysis software.

PEDV has become the most important intestinal pathogen in swine in China (33). Many studies of the mechanism of PEDV infection and effective vaccines have been published. However, the variations of the virus and its co-infections with other enteric viruses, had contributed to the poor control of PEDV infection. In order to better understand the prevalence of the co-infections in Shanghai, an epidemiological investigation of porcine diarrhea viruses was carried out in this study. It was reported that the co-infection of PEDV and PBoV was more prevalent in diarrhea samples than non-diarrhea samples (34). A recent study showed that 27% of samples had PEDV infection alone, whereas the remaining 73% of samples exhibited two to nine pathogens (35). According to this survey, single infection with PEDV occurred in only 6.56% of samples (34 out of 753), while most of the samples involved co-infections. Except for the co-infections with PEDV, various other types of co-infections existed in the study. It was considered that animals co-infected with more than one enteric virus experienced increased intestinal epithelium damage and/or viral replication, which resulted in more severe diarrhea (27). Forty samples of diarrhea in piglets in Sichuan Province were tested and five samples (12.5%) of multiplex infections of PKoV, PAstV, and PToV were identified (6). Chang et al. (30) tested 165 samples obtained from 42 pig farms, and reported that 2 of 42 pig farms were infected with PEDV and TGEV, accounting for 4.76%. Furthermore, seven pig farms were infected with PEDV and PoRV, accounting for 16.67%, and two pig farms were infected with three viruses, accounting for 4.76%. This was consistent with the present results, in which there were serious multiplex infections in these pathogens. Among the 11 enteric viruses, 53.27% (114/214) of samples had at least two pathogens, and 31.78% (68/214) of samples had three different pathogens, while the positive rate of quadruple- and quintuple-infection was 11.68% (25/214) and 1.87% (4/214), respectively. Notably, co-infection with six viral diarrhea pathogens was identified in three samples (1.40%). However, no new vaccines for diarrhea pathogens had been developed and applied to pig farms. Furthermore, there have been instances of co-infections in sows, even though these are usually asymptomatic. This may explain the persistence of viruses within the herd, and facilitation of vertical transmission.

PKoV can infect pigs of all ages and varieties with prevalence of ranges from 19.3 to 99.0% in different countries (17). Since the first report of PKoV in Hungary (13) and China (15), it has been confirmed that PKoV was widely present in several countries, and plays an important role in diarrhea outbreak in pigs (14, 16, 36–38). The statistical analysis of the PKoV positive rate between diarrheic and healthy pigs, as well as a survey for other enteric pathogens in diarrheic pigs, suggested that PKoV may play a role as a causative agent of gastroenteritis in pigs (37). Recent studies have revealed the genetic diversity and possible pathogenic role of PKoV in conjunction with other pathogens in piglets (30, 37). PKoV has also been linked to porcine diarrhea although its pathogenesis remains unclear (16). It was reported that piglets with diarrhea shed more PKoV than healthy individuals during the late-nursing stage (6–21 days old) (39), which accounted for the high positive rate of PKoV. Based on the rates of infection documented in this study, PKoV exhibited the highest infection rate (38.65%), and high co-infection rates ranging from 1.75 to 28.07%, suggesting a high prevalence of co-infections in the sampled regions. Either mono-infection or co-infections of PKoV were common in pigs in local China, therefore, further investigations should be conducted to research its characteristics and pathogenic mechanism. Interestingly, co-infections with PEDV and PKoV accounted for 31.87% (29/91) of the PEDV positive samples, which was similar with the frequency of infection with PEDV alone (37.36%, 34/91). These data suggested that PKoV had a potential role in PEDV-induced diarrhea symptoms, which was consistent with the previous study (39). The high prevalence of co-infection, particularly PKoV and PEDV, is a cause for concern and should be seriously considered.

Although diarrhea pathogens were frequently identified in the winter, diarrhea infection occurred throughout the year in pig farms. Since the pathogen composition of different farms varied and multiple infections emerged more frequently, clinical control should be based on pathogen monitoring. In addition to viral factors, bacteria or the interaction between bacteria and viruses might also contribute to the complexity of the etiology of porcine diarrhea. A single pathogen might not be the main cause of diarrhea, however, the unique relationship among different pathogens needs further research. Although the specific mechanism of the diarrhea pathogen for porcine diarrhea disease remains complex, the potential hazards cannot be ignored, and attention should be given to the relevant vaccines in order to prepare for the outbreak of a new round of porcine diarrhea disease in advance.

## Conclusions

Here we provided a Luminex xTAG multiple detection method for 11 kinds of porcine viral diarrhea pathogens in clinic, which was more sensitive and specific than general multiplex PCR method. Furthermore, the surveillance confirmed that variant enteropathogenic viruses were leading etiologic agents of porcine diarrhea, either mono-infections or co-infections of PKoV were common in pigs in local China, but PEDV was still the key pathogen and multiple pathogens synergistically complicated the infection status, suggesting that controlling porcine diarrhea might be more complex than previously thought. The study provides a better understanding of viruses that cause diarrhea in piglets, which will aid in better preventing and controlling epidemics of viral porcine diarrhea. Furthermore, attention should be given to the relevant vaccines, and the expansion of the vaccine reserve, in order to prepare for the outbreak of a new round of porcine diarrhea disease in advance.

## Data Availability Statement

The original contributions presented in the study are included in the article/Supplementary Materials, further inquiries can be directed to the corresponding author/s.

## Ethics Statement

The animal study was reviewed and approved by the animal monitoring committee of Shanghai Academy of Agricultural Science.

## Author's Note

This manuscript has been released as a pre-print at https://www.researchgate.net/publication/342085863 (40).

## Author Contributions

HL designed the research. YS, BL, JT, and JC performed the research. YS, BL, and HL analyzed data and wrote the paper. All authors contributed to the article and approved the submitted version.

## Conflict of Interest

The authors declare that the research was conducted in the absence of any commercial or financial relationships that could be construed as a potential conflict of interest.

## Supplementary Material

Supplementary Figure 1 The establishment of the detection method for singular pathogen. **(A)** The result of singular PCR based on the standard plasmid. M: DL500 DNA Marker; 1: PSaV; 2: negative control; 3: PTV; 4: negative control; 5: PSV; 6: negative control; 7: PKoV; 8: negative control; 9: PAstV; 10: negative control; 11: PDCoV; 12: negative control; 13: PToV; 14: negative control;15: TGEV;16: negative control; 17: PEDV; 18: negative control; 19: BVDV; 20: negative control; 21: PoRV;22: negative control. **(B)** The detection of singular Luminex xTAG reaction. Each column contains a negative control.

Supplementary Figure 2 The comparison of the sensitivity between the Luminex xTAG detection method and traditional RT-PCR method. The standard plasmids were 10-fold multiplicationally diluted to be detected as the templates. **(A)** The sensitivity of traditional RT-PCR method for seven kinds of diarrhea viruses. M: DL1000 DNA Marker; 1–5: 10^−1^-10^−5^ fold dilution template, respectively; 6: negative control. **(B)** The sensitivity of traditional RT-PCR method for four kinds of diarrhea viruses. M: DL1000 DNA Marker; 1–7: 10^0^-10^−5^ fold dilution template, respectively; 8: negative control. **(C)** The sensitivity of the Luminex xTAG detection method for seven kinds of diarrhea viruses. **(D)** The sensitivity of the Luminex xTAG detection method for four kinds of diarrhea viruses.

## Abbreviations

PKoV: Porcine kobuvirus
PAstV: porcine astrovirus
PEDV: porcine epidemic diarrhea virus
PSV: porcine sapelovirus
PSaV: porcine sapovirus
PTV: porcine teschovirus
PDCoV: porcine deltacoronavirus
PoRV: porcine rotavirus
TGEV: transmissible gastroenteritis virus
PToV: porcine torovirus
BVDV: bovine viral diarrhea virus
PBS: phosphate-buffered saline
DEPC: diethylpyrocarbonate
PCR: polymerase chain reaction
ELISA: enzyme linked immunosorbent assay
RT-PCR: reverse transcription-polymerase chain reaction
MFI: mean fluorescence intensity
SAPE: Streptavidin-phycoerythrin.
