# Supplementary figures and images for: The Complex Co-infections of Multiple Porcine Diarrhea Viruses in Local Area Based on the Luminex xTAG Multiplex Detection Method
Source: Front Vet Sci. 2021 Jan 28;8:602866. doi: 10.3389/fvets.2021.602866 (PMC7876553; doi:10.3389/fvets.2021.602866)

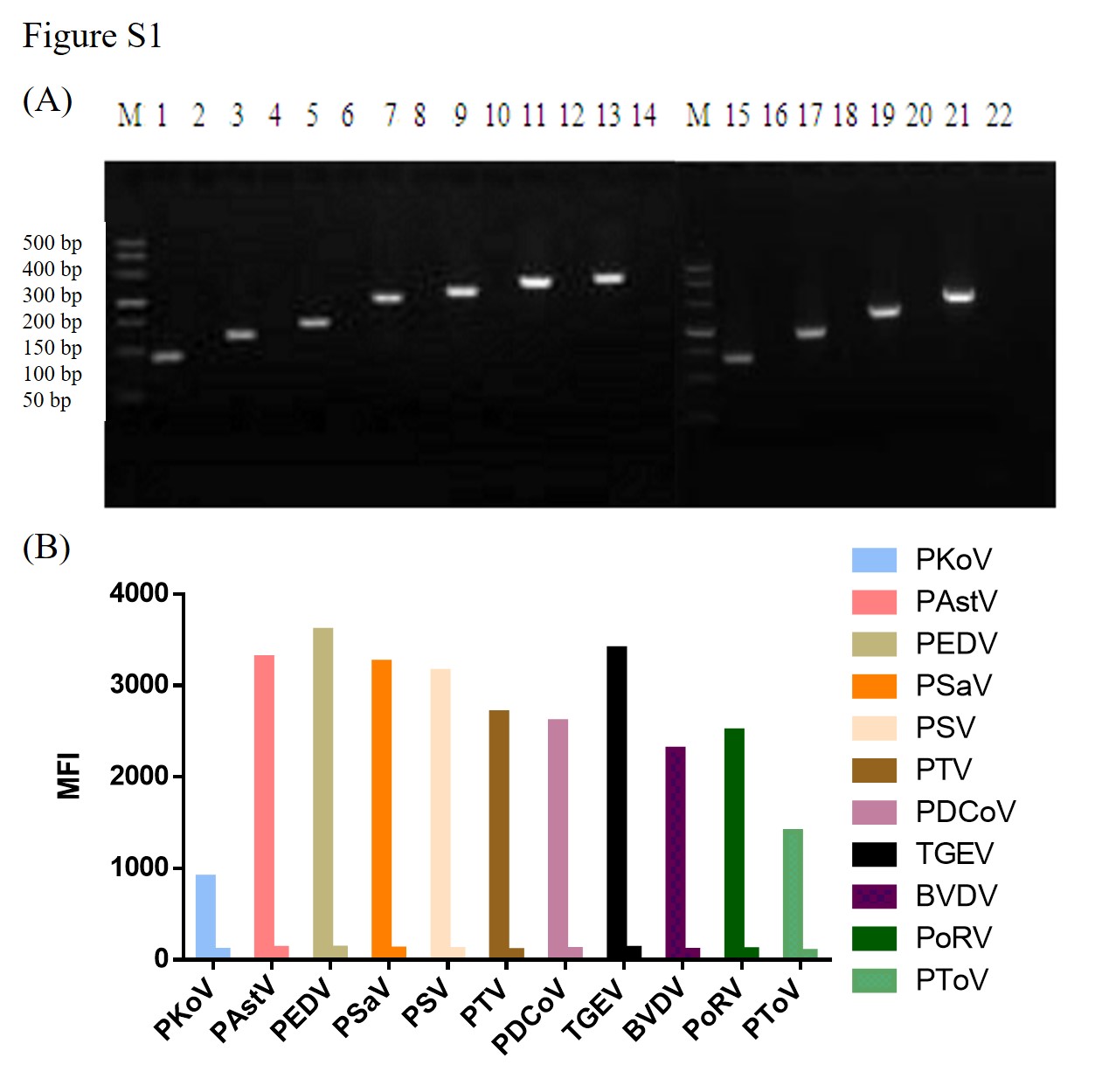

Supplement: Supplementary Figure 1 — The establishment of the detection method for singular pathogen. (A) The result of singular PCR based on the standard plasmid. M: DL500 DNA Marker; 1: PSaV; 2: negative control; 3: PTV; 4: negative control; 5: PSV; 6: negative control; 7: PKoV; 8: negative control; 9: PAstV; 10: negative control; 11: PDCoV; 12: negative control; 13: PToV; 14: negative control;15: TGEV;16: negative control; 17: PEDV; 18: negative control; 19: BVDV; 20: negative control; 21: PoRV;22: negative control. (B) The detection of singular Luminex xTAG reaction. Each column contains a negative control. [file Image_1.JPEG]

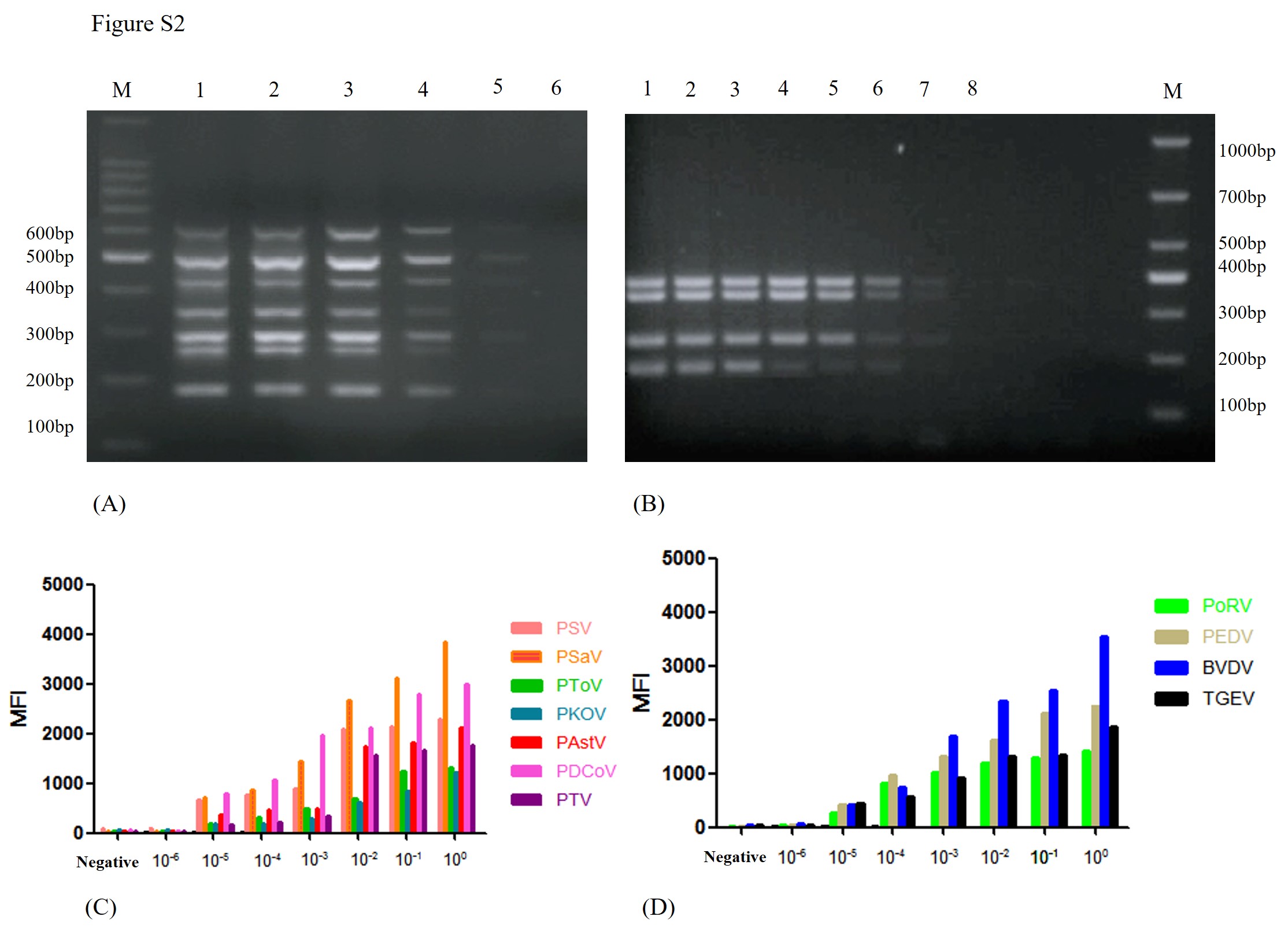

Supplement: Supplementary Figure 2 — The comparison of the sensitivity between the Luminex xTAG detection method and traditional RT-PCR method. The standard plasmids were 10-fold multiplicationally diluted to be detected as the templates. (A) The sensitivity of traditional RT-PCR method for seven kinds of diarrhea viruses. M: DL1000 DNA Marker; 1–5: 10−1-10−5 fold dilution template, respectively; 6: negative control. (B) The sensitivity of traditional RT-PCR method for four kinds of diarrhea viruses. M: DL1000 DNA Marker; 1–7: 100-10−5 fold dilution template, respectively; 8: negative control. (C) The sensitivity of the Luminex xTAG detection method for seven kinds of diarrhea viruses. (D) The sensitivity of the Luminex xTAG detection method for four kinds of diarrhea viruses. [file Image_2.JPEG]
